# NOL12 as an Oncogenic Biomarker Promotes Hepatocellular Carcinoma Growth and Metastasis

**DOI:** 10.1155/2022/6891155

**Published:** 2022-06-02

**Authors:** Jianfeng Huang, Weibiao Kang, Shubo Pan, Changjun Yu, Zhigang Jie, Changyu Chen

**Affiliations:** ^1^Department of Gastrointestinal Surgery, The First Affiliated Hospital of Nanchang University, Nanchang, 330006 Jiangxi Province, China; ^2^Department of General Surgery, The First Affiliated Hospital of Anhui Medical University, 218 Jixi Avenue, Hefei, 230022 Anhui, China; ^3^Department of General Surgery, The Second Affiliated Hospital of Anhui Medical University, No. 678 Furong Road, Hefei, Anhui 230022, China

## Abstract

Hepatocellular carcinoma (HCC) is a common malignancy with a poor prognosis worldwide. However, the pathogenesis of HCC remains poorly understood. In this study, we found that NOL12 was significantly overexpressed in independent HCC datasets from TCGA database. We confirmed that the expression level of NOL12 was upregulated in human HCC tissues and cell lines by RT-qPCR. High expression of NOL12 is associated with worse reduced overall survival (OS), high pathological grade, node metastasis, and advanced clinical stage in patients with HCC. Moreover, knockdown of NOL12 dramatically inhibits the proliferation and metastasis of HCC cells in vitro and in vivo. CIBERSORTx analysis revealed that twelve types of tumor-infiltrating immune cells (TICs) are correlated with NOL12 expression. The risk signature based on 8 NOL12-related genes is an independent prognostic factor for patients with HCC. The OS rate of patients in the low-risk score group was better than that in the high-risk score group. In addition, the total tumor mutation burden (TMB) in the high-risk score group increased significantly, and the risk scores could be used as an alternative indicator of immune checkpoint inhibitor (ICI) response. In conclusion, our findings indicated that NOL12 might be involved in the progression of HCC and can be used as a potential therapeutic target. Moreover, the NOL12-related risk signature may have predictive relevance with regard to ICI therapy.

## 1. Introduction

Liver cancer has become the fourth largest cause of cancer-related death in the world, with the sixth highest incidence of all cancers. It is estimated that approximately 1 million people will die of liver cancer each year [1, 2]. Hepatocellular carcinoma (HCC), as the most common primary malignant tumor of the liver, progresses rapidly, and patients are usually diagnosed at an advanced stage. At present, the treatment of HCC is limited and mainly depends on hepatectomy, chemotherapy, and immunotherapy [3]. However, due to early metastasis, postoperative recurrence, and the emergence of drug resistance and other factors, the 5-year survival rate of patients has not been significantly improved [4, 5]. Moreover, there is still a lack of effective methods to predict the prognosis of patients and provide individualized treatment [6]. Therefore, it is urgent to find more reliable diagnostic biomarkers and develop new indicators to predict patient survival to provide individualized treatment strategies.

The transcription and regulation of the human genome are a sophisticated process, in which RNA-binding proteins participate and play an important role [7, 8]. In recent years, an increasing number of studies have found that RNA-binding proteins are also involved in tumor progression and immune regulation [9–12]. Nucleolar protein 12 (NOL12), a multifunctional RNA-binding protein, has been found to be related to genomic stability, DNA damage repair, and apoptosis [13]. Pinho et al. [14] reported that NOL12 can affect the cell cycle and eventually lead to cell senescence in an RPL11-dependent manner. In addition, some studies have shown that NOL12 may be a potential oncogene for a variety of tumors and may be related to the prognosis of patients [15, 16].

Tumor microenvironment (TME) is an important factor affecting the progression and treatment of HCC [17]. Some studies have shown that, when all kinds of immune cells in the TME reach a certain balance, they will spur immune activation, thus promoting the occurrence of immune escape [18]. Increasing evidence has shown that tumor-infiltrating immune cells (TICs) affect the biological behavior of HCC cells and ultimately affect the prognosis of patients [19, 20]. In addition, the results of some latest clinical trials and medical studies showed that immunotherapy or combined with immunotherapy was more beneficial to patients with advanced HCC [21, 22]. For those patients with previously untreated unresectable HCC, the overall survival and disease-free survival after treatment with combination ICIs were significantly longer, and patients were more likely to undergo follow-up surgery [23, 24]. However, whether NOL12 promotes the progression of hepatocellular carcinoma, whether it affects the immune infiltration of HCC, and whether it can be used as a predictor of ICIs therapy have not been reported.

In this study, we first confirmed that the expression of NOL12 is upregulated in HCC through TCGA database, and it is associated with poor prognosis. Second, through CIBERSORTx and functional enrichment analysis, we found that NOL12 is related to a variety of TICs and tumor-related signaling pathways. In addition, we developed a risk signature based on NOL12-related genes and established a nomogram that can independently predict the clinical outcome of HCC. Patients with different risk scores have been shown to have different therapeutic potentials for immune checkpoint inhibitors (ICIs). Finally, we determined the upregulated expression of NOL12 in HCC and the tumorigenic roles of NOL12 through a series of in vitro and in vivo experiments. Overall, our results indicated that NOL12 can act as a novel prognostic biomarker and a potential therapeutic target for HCC.

## 2. Materials and Methods

### 2.1. Data Acquisition and Processing

The RNA transcriptome dataset, the corresponding clinical data, and the mutation profiling from TCGA (https://tcga-data. http://nci.nih.gov/tcga/) database for the liver hepatocellular carcinoma (LIHC) project were downloaded. The limma package for R software was employed to further process RNA expression data. When combining clinical information, missing and incomplete samples were deleted. The remaining TCGA dataset contains 370 tumor samples and 50 normal samples for further analysis.

### 2.2. TIC Profile

The abundant distribution of TICs in all tumor samples was estimated using the CIBERSORTx [25] computational method, and the corresponding R package was used to visualize them.

### 2.3. Functional Enrichment and Coexpression Analysis

We used Gene Ontology (GO) and Kyoto Encyclopedia of Genes and Genomes (KEGG) enrichment analysis with Gene Set Enrichment Analysis (GSEA) software to explore the potential biological function of NOL12 in HCC. Pathways with *P* < 0.05 were considered significantly enriched. The visualization was performed by the ggplot2 package. In addition, a protein-protein interaction (PPI) network highly related to the NOL12 encoded protein was obtained using the Search Tool for the Retrieval of Interacting Genes (STRING; http://string-db.org), with the highest confidence (0.9).

### 2.4. NOL12-Related Risk Signature Construction

We screened prognosis-related coexpressed genes by univariate Cox regression analysis. To avoid overfitting, all the selected genes were involved in the subsequent least absolute shrinkage and selection operator (LASSO) penalized Cox regression analysis. The risk score for each patient with HCC was calculated based on the following formula: risk score = ∑(Coefi∗Expxi), where Expxi represents each gene expression and Coefi represents the coefficient of each gene.

### 2.5. Validation of the Risk Signature

All TCGA LIHC patients were randomly divided into training sets (186) and testing sets (184). The patients in the training and testing sets were further divided into high- and low-risk groups, with the median risk score as the cutoff value. To evaluate the accuracy of the risk signature, Kaplan-Meier survival analysis and time-dependent receiver operating characteristic (ROC) curves were performed on the training sets and testing sets. The complete dataset with corresponding clinical information was used for subsequent stratified survival analysis. To identify the independence of the risk signature, univariate Cox regression analysis between the risk signature and the clinicopathological features was performed, and a *P* < 0.05 was considered to be a significant independent prognostic factor.

### 2.6. Construction and Assessment of a Nomogram

The nomogram was constructed based on the risk score and independent clinical factors by using the “regplot” R package to quantify risk evaluation and predict the clinical outcomes of patients with HCC. The calibration plot was applied to assess the accuracy of the nomogram for predicting the 1-, 3-, and 5-year overall survival of patients with HCC. Decision curve analysis (DCA) was used to assess the clinical practicability of the nomogram.

### 2.7. TMB and ICI Analysis

The somatic variant data of patients with HCC were analyzed and visualized by package maftools. Then, difference, correlation, and survival analyses were used to evaluate the effects of TMB and risk scores on the prognosis of patients with HCC.

In addition, we also compared the expression levels of common immune checkpoints in the high-risk score and low-risk score groups and the correlation between immune checkpoint expression level and risk score.

### 2.8. Cell Lines and Culture

Human liver cancer cells (BEL-7404, Hep3B, Huh-7, and HepG2) and human liver cell L02 were obtained from Shanghai Cell Bank (Shanghai, China) or ATCC (the American Type Culture Collection). All the cell lines used in this study were approved by the Ethics Committee of the First Affiliated Hospital of Nanchang University. All cell lines were cultured in 90% DMEM and 10% FBS at 37°C in a 5% CO_2_ incubator humidified atmosphere as recommended.

### 2.9. RT-qPCR

The mRNA levels of NOL12 in fresh liver cancer tissues and liver cancer cell lines were examined using RT quantitative PCR (RT-qPCR). RNA was reverse-transcribed into cDNA and subjected to PCR reactions using the EasyScript One-Step RT-PCR kit (TRAN, AE311-03). GAPDH was used as control. The primers used in this study were as follows: NOL12 (F: GCCGCAAAAAGGTCAAGAGG, R: CCCAAAAACCTGCCCTTGTG) and GAPDH (F: CTGCCTCTAC TGGCGCTG, R: GGTCAGGTCCACCACTGAC). The relative expression of NOL12 mRNA was normalized to the expression level of mRNA using the 2−*Δ*Ct method.

### 2.10. Lentivirus Cell Transfection

Lentiviral vectors encoding short hairpin RNAs (shRNAs) that target NOL12 were purchased from GenePharma (Shanghai, China). NOL12 shRNAs were transfected into Huh-7 and HepG2 cells according to the manufacturer's instructions. In order to obtain stable cell lines, the transduced cells were selected in the medium containing puromycin (5.0 *μ*g/mL).

### 2.11. Cell Functional Assays

The MTT assay was used to evaluate cell viability. The steps were as follows: 1000 cells were placed in a 96-well plate. From day 1 to day 5, 20 *μ*L of MTT solution (5 mg/mL) was added to each well, and the wavelength of 570 nm was measured with a 96-well plate reader. For the cell colony formation assay, 1000 HepG2 and Huh-7 cells/well from the control and experimental groups were inoculated into 6-well plates, and colony formation was detected 10 days later.

In the migration assay, 5‐10 × 10^4^ HepG2 and Huh-7 cells were added to the top 8 *μ*m chamber without Matrigel, while in invasion assays, Matrigel was added. After 24-48 h of incubation, the inserts were rinsed with PBS and stained with 0.1% crystal violet solution for 5 min. Images were taken with an Olympus IX-70 microscope.

### 2.12. Xenograft Assays

All experiments involving animal subjects were in accordance with the institutional animal welfare guidelines and approved by the First Affiliated Hospital of Nanchang University. Five-week-old male nude mice maintained in the Public Platform Laboratory Animal Center were used in xenograft assays. A total of 5 × 10^6^ Huh-7 cells were mixed with the same volumes of Matrigel and subcutaneously injected into the flanks or tail veins of each animal (6 animals per experimental group). The volumes of tumors were measured every 3 days when tumor masses were identified. Tumor volume = [(length) × (width) × (width)]/2. All mice were sacrificed by injection of a deadly dose of pentobarbital sodium (150 mg/kg) at the 28th day after tumor cell injection, and cervical dislocation was performed to confirm death. Subsequently, the mice were dissected and tumors were removed and weighed.

### 2.13. Immunohistochemistry

Tissue samples were paraffin embedded, sliced, and used for immunohistochemical testing according to standard procedures. Antibodies against NOL12 (1 : 100, A302-733A, Thermo Fisher Scientific, USA) and antibodies against Ki-67 (1 : 100, ab15580, Abcam, UK) were used.

### 2.14. Statistical Analysis

Data between two groups were examined using a two-tailed paired Student *t*-test or ANOVA (Bonferroni post hoc test). Correlation analysis was performed using the Pearson chi-square test or Spearman rank correlation test. All statistical analyses were performed by GraphPad Prism 7 or R (4.0.0); *P* values < 0.05 denoted statistically significant differences.

## 3. Results

### 3.1. NOL12 Is Upregulated in HCC and Associated with a Poor Prognosis

**Figure 1:**
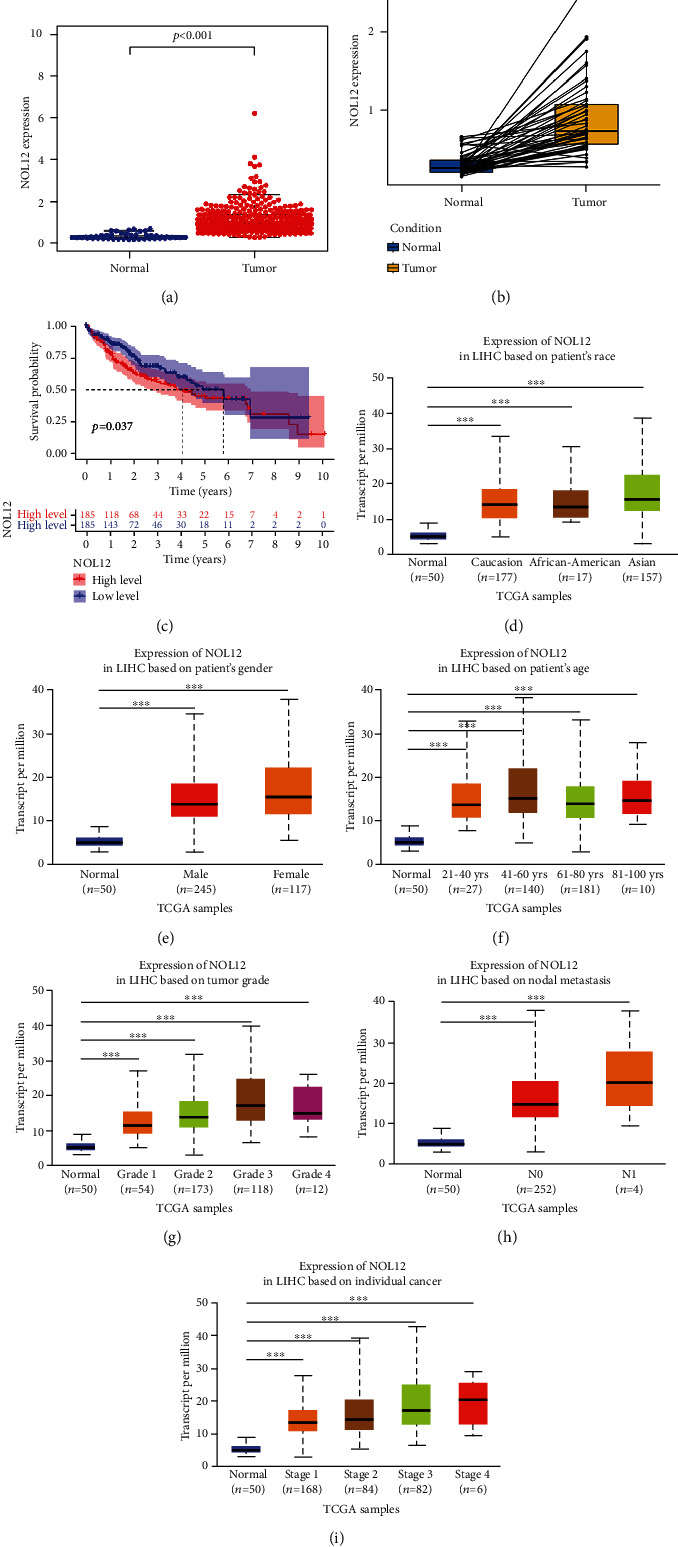
NOL12 is upregulated in HCC, and its expression is associated with poor survival in HCC patients. (a) Differential and (b) paired differential analysis of NOL12 in normal liver tissue and HCC tissue (*P* < 0.01). (c) Kaplan-Meier curve was used to analyze the overall survival (OS) rate of patients with high and low expression of NOL12 (*P* = 0.037). The correlation of NOL12 expression with clinicopathological characteristics: (d) race; (e) gender; (f) age; (g) grade; (h) metastasis status; (i) stage. ^∗∗∗^*P* < 0.001.

First, we downloaded the transcriptome data of LIHC and the corresponding clinical data from TCGA database, including 50 normal and 370 tumor samples. To evaluate the significance of NOL12 in HCC, these datasets were used to study the expression of NOL12 in HCC. The results showed that the mRNA expression of NOL12 in HCC tissues increased significantly (Figure 1(a)). This result was also fully verified in the paired analysis of HCC tissue and adjacent normal tissue in the same patient (Figure 1(b)). In addition, we found that the overall survival rate of the NOL12 high expression group was lower than that of the low expression group, which was statistically significant (Figure 1(c)). We further examined the correlation of NOL12 with the clinicopathologic features of HCC. In a subgroup analysis based on race, sex, age, grade, metastasis status, and stage in the UALCAN database, the transcription level of NOL12 in patients with LIHC was significantly higher than that in normal controls, and the higher the expression of NOL12 was, the later the tumor grading and staging (Figures 1(d)–1(i)). The above results show that NOL12 may be a potential diagnostic marker for HCC.

### 3.2. Correlation of NOL12 with the Proportion of TICs

**Figure 2:**
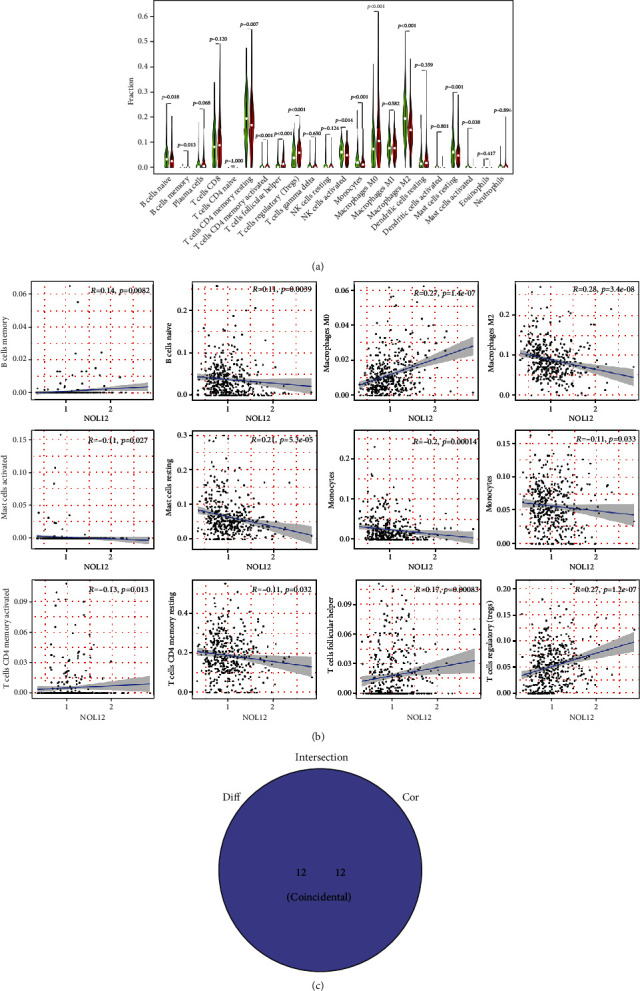
Correlation of NOL12 with the proportion of TICs. (a) Violin plot displaying the differentially infiltrated immune cells between LIHC tumor samples with high or low NOL12 expression relative to the median NOL12 expression level. Green represents the NOL12 low expression group and red represents the NOL12 high expression group. (b) Scatter plot showed that 12 kinds of immune cells correlated positively or negatively with the expression of NOL12 (*P* < 0.05). (c) Venn diagram displaying twelve kinds of immune cells that correlated with NOL12 expression codetermined by difference and correlation tests shown in violin and scatter plots, respectively.

**Figure 3:**
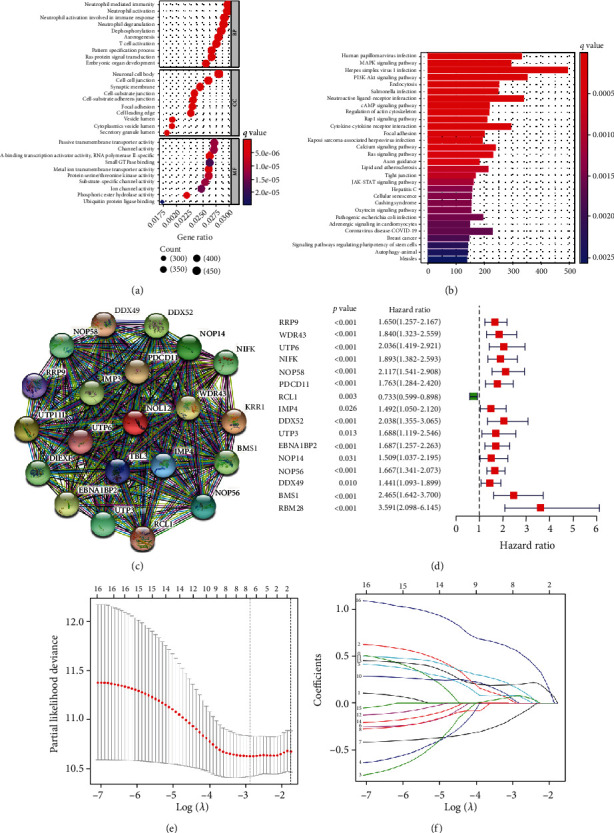
Gene set enrichment analysis and construction of a risk signature based on NOL12-related genes. (a) GO and (b) KEGG enrichment analysis. (c) The network view provides the predicted association network of proteins that are highly related to the protein product of NOL12. (d) Forest map showing 16 genes strongly associated with HCC prognosis identified by the univariate cox regression method. (e) Partial likelihood deviance of different numbers of variables revealed by the LASSO regression model. (f) LASSO coefficient profiles of the NOL12-related genes.

We next systematically described the pattern of tumor-infiltrating immune cells (TICs) by processing the signature gene expression profile in HCC with the CIBERSORTx algorithm. The results of Supplementary Figure 1 show the distribution landscape and correlation of infiltrating immune cells in LIHC specimens. According to the results from the difference and correlation analyses, we concluded that NOL12 might regulate the immune activity of the TME in HCC mainly through twelve kinds of TICs (Figure 2). Among them, seven kinds of TICs were negatively correlated with NOL12 expression, including naïve B cells, resting CD4+ T cell memory, activated NK cells, monocytes, M2 macrophages, resting mast cells, and activated mast cells. Five kinds of TICs were positively correlated with NOL12 expression, including memory B cells, M0 macrophages, activated CD4+ T cell memory, follicular helper T cells, and regulatory T cells. In addition, the results from GO enrichment analysis indicated that the functions of NOL12 correspond to immune-related activities (Figure 3(a)). These results suggested that the level of NOL12 might be a key indicator to reflect the state of the TME.  Figure 3(b) shows that a variety of tumor-related signaling pathways were enriched, including the MAPK, PI3K-Akt cAMP, and Ras signaling pathways.

### 3.3. Construction of a NOL12-Related Gene Risk Signature

We constructed a PPI network of NOL12 using the Search Tool for the Retrieval of Interacting Genes (STRING) database, with the highest confidence (0.9) (Figure 3(c)).

We obtained the 20 genes most closely related to NOL12 and then performed univariate Cox regression analysis to further evaluate the relationship between these coexpressed genes and the survival of patients with HCC. The results showed that 16 NOL12 coexpressed genes were closely related to the prognosis of HCC (Figure 3(d)). All patient samples were randomly assigned to a training set (*n* = 186) or a testing set (*n* = 184). Least absolute shrinkage and selection operator (LASSO) proportional Cox regression analysis was performed to construct an eight-gene risk signature model (Figures 3(e) and 3(f)). The risk score of each patient in the training and testing sets was calculated as follows:risk score = (expWDR43∗0.00322) + (expNOP58∗0.08182) + (expRCL1∗−0.13789) + (expUTP3∗0.01855) + (expEBNA1BP2∗0.16428) + (expNOP56∗0.19824) + (expBMS1∗0.08048) + (expRBM28∗0.55925).

### 3.4. Prognostic Value of the NOL12-Related Gene Risk Signature

**Figure 4:**
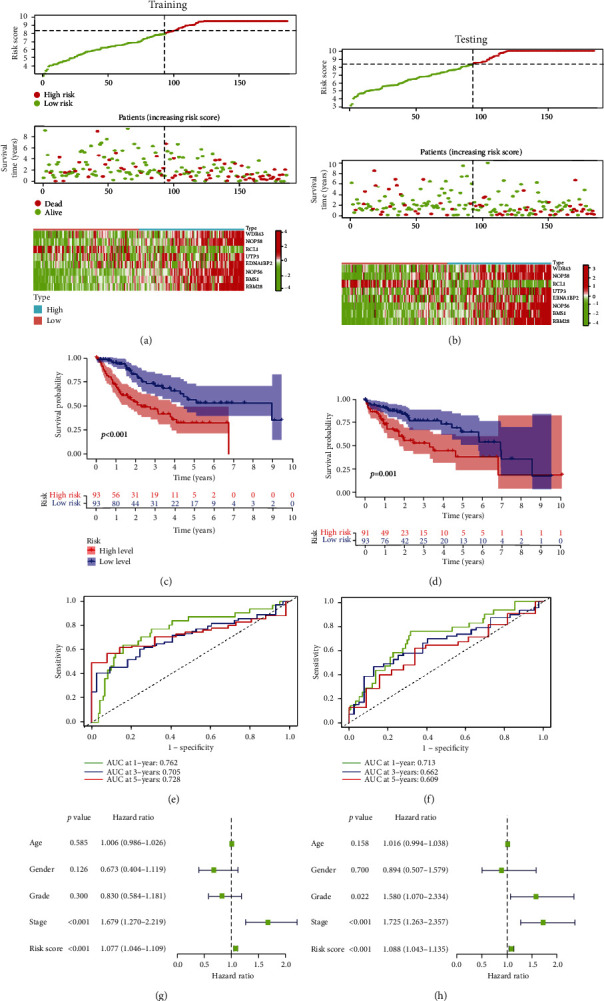
Validation of risk signatures of NOL12-related genes in the training and testing cohorts. (a, b) Distribution of risk score, survival status, and gene expression among patients in the training and testing cohorts. (c, d) The Kaplan–Meier curves of the high-risk and low-risk groups in the training and testing cohorts were compared. (e, f) ROC curves showing the predictive efficiency of the risk score for the 1-year, 3-year, and 5-year survival rate in the training and testing cohorts. (g, h) Univariate Cox regression analysis of prognostic factors in the training and testing cohorts.

The patients in the training and testing sets were further divided into high- and low-risk groups, with the median risk score as the cutoff value. The results showed that in both the training and testing sets, the number of deaths in the high-risk group was significantly higher than that in the low-risk group (Figures 4(a) and 4(b)). The results of K-M survival analysis also confirmed that the prognosis of the low-risk group was better than that of the high-risk group (Figures 4(c) and 4(d)). Time-dependent receiver operating characteristic (ROC) curves were generated to estimate the efficacy of the risk signature for predicting the survival of patients with HCC. The areas under the curve (AUCs) of the 1-, 3-, and 5-year risk scores were 0.762, 0.705, and 0.728 in the training sets, respectively. In the testing sets, the AUC values were 0.713, 0.662, and 0.609 at the 1-, 3-, and 5-year time points, respectively (Figures 4(e) and 4(f)). In addition, to further evaluate the value of the risk signature in clinical application, we conducted a stratified survival analysis of patients with different clinicopathological features, including age, sex, grade, T stage, N stage, M stage, and clinical stage. For different stratifications, patients in the low-risk group had a higher overall survival rate than those in the high-risk group (Supplementary Figure 2). These results indicated that the NOL12-related risk signature can accurately predict the prognosis of patients with HCC without considering clinical factors. Univariate Cox regression analyses also showed that the risk score was an independent prognostic factor for patients with HCC (Figures 4(g) and 4(h)).

### 3.5. Construction of a Nomogram and Evaluation of Its Prediction Ability

**Figure 5:**
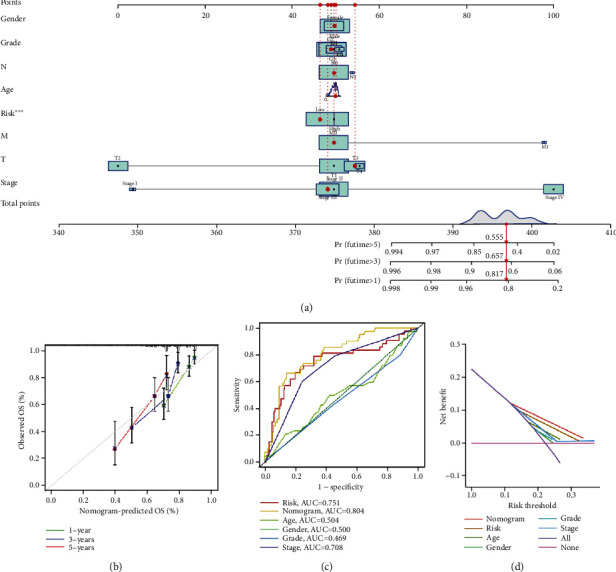
Construction of nomogram and evaluation of its prediction ability. (a) A nomogram was constructed based on independent factors to predict the 1-year, 3-year, and 5-year overall survival in HCC patients. (b) Calibration curve for the prediction of 1-year, 3-year, and 5-year survival in HCC patients. (c) ROC curve analysis of different clinical factors in complete dataset. (d) Decision curve analysis of the nomogram. *X*-axis represents the percentage of threshold probability, and *Y*-axis represents the net benefit.

To provide a quantitative method with clinical value to predict the probability of 1-, 3-, and 5-year OS in HCC, we constructed a nomogram based on the independent prognostic factors (Figure 5(a)). The total scores were calculated according to the points of all variables in the nomogram, and then, the 1-, 3-, and 5-year survival rates of each patient with HCC could be predicted by drawing a vertical line from the total points to the survival prediction axis. The prediction values of the 1-, 3-, and 5-year nomograms in the calibration plot were close to the 45-degree line in the complete dataset, which indicates that the nomogram demonstrates good prediction ability (Figure 5(b)). The ROC curves showed that, compared with other clinical factors related to prognosis, the nomogram had the highest accuracy in predicting HCC survival, with an AUC of 0.804 (Figure 5(c)). In addition, decision curve analysis (DCA) was performed to evaluate the net clinical benefits of multiple prognostic factors (Figure 5(d)). The results showed that using a nomogram to predict survival probability can bring more benefits to patients.

### 3.6. Estimation of TMB and ICIs by the Risk Signature

**Figure 6:**
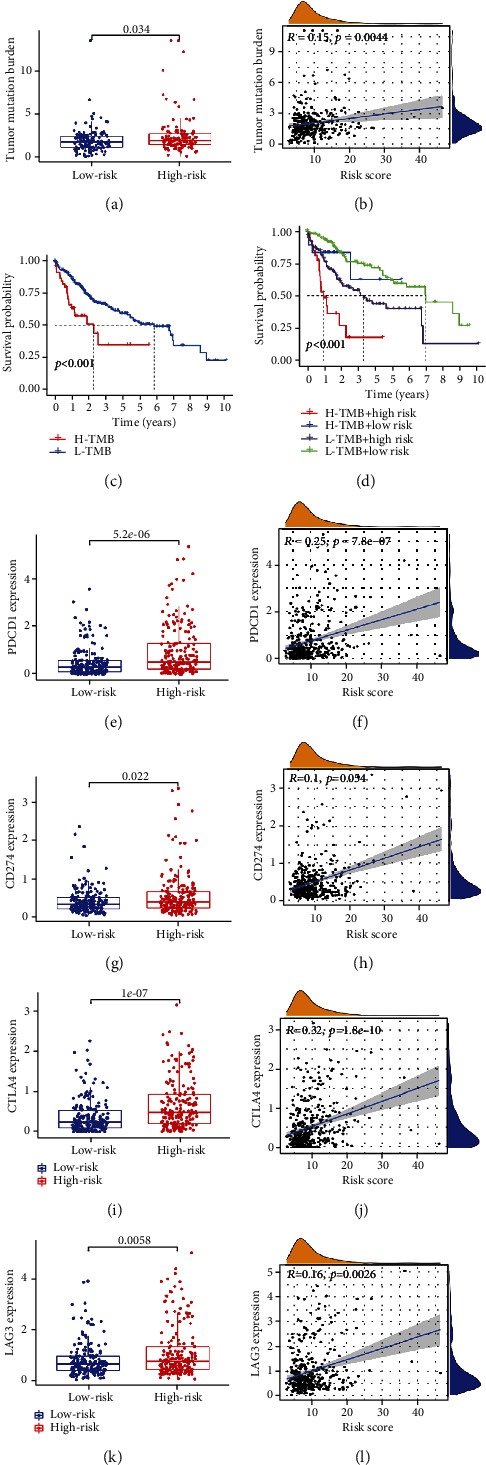
Estimation of tumor mutation burden (TMB) and immune checkpoint inhibitors by risk signature. (a, b) Analysis of the difference and correlation between tumor mutation burden and risk scores. (c, d) The effect of tumor mutation burden and risk scores on the overall survival of HCC patients. High risk scores were correlated with upregulated (e, f) PD-1 and (g, h) PD-L1, (i, j) CTLA4, and (k, l) LAG3 in HCC patients.

We analyzed the mutation profiles of each LIHC patient and associated the patient's risk score with TMB for difference and correlation analyses. The results showed that the level of TMB in the high-risk group was higher than that in the low-risk group and was positively correlated with the risk score (Figures 6(a) and 6(b)). Subsequently, we performed a K-M survival analysis to evaluate the effects of high and low TMB and risk scores on the prognosis of patients with HCC. As shown in Figures 6(c) and 6(d), the survival rate of patients with h-TMB was significantly lower than that of patients with l-TMB. Among all the groups, the survival rate of patients with h-TMB+h-risk was the worst, while that of patients with l-TMB+l-risk was the best. Since patients with elevated TMB tend to benefit more from ICI treatment, we further investigated the relationship between ICIS and risk scores. The results showed that the expression levels of PD-1, PD-L1, CTLA-4, and LAG3 in the high-risk group were significantly higher than those in the low-risk group and were positively correlated with the risk score, indicating that the high-risk group could benefit more from ICI treatment (Figures 6(e)–6(l)).

### 3.7. Knockdown of NOL12 Inhibits the Proliferation of HCC Cells

**Figure 7:**
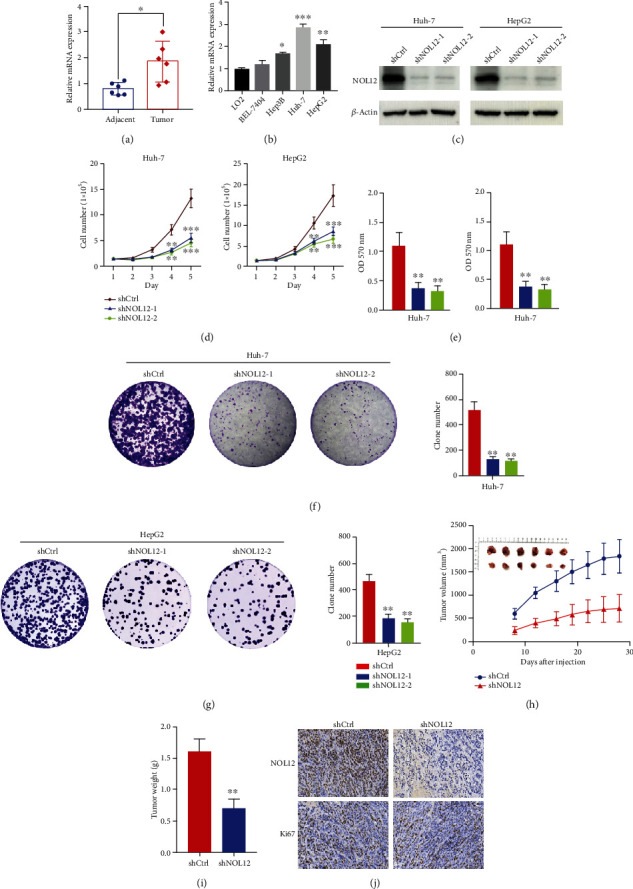
NOL12 knockdown inhibited cell proliferation of HCC cells in vitro and in vivo. The expression levels of NOL12 in 12 pairs of fresh adjacent nontumor tissues and liver cancer tissues (a) and HCC cell lines (b) were detected by RT-qPCR. (c) Endogenous NOL12 proteins were efficiently silenced (shNOL12-1, shNOL12-2) in Huh-7 and HepG2 cells. Actin was used as control. (d, e) MTT assays were performed to determine the proliferation of HCC cells transduced with the NOL12 shRNA lentivirus. (f, g) Cell colony formation assays were performed in HCC cells. (h) Tumor volume was monitored in xenograft samples from stable NOL12 knockdown Huh-7 cells or shCtrl. (i) Final weight of tumor masses obtained from stable NOL12 knockdown Huh-7 cells or shCtrl was compared. (j) The proportion of Ki67-expressing cells in xenograft tumors was examined by immunohistochemistry. ^∗^*P* < 0.05, ^∗∗^*P* < 0.01, and ^∗∗∗^*P* < 0.001.

To further confirm the upregulated expression of NOL12 in HCC, RT-qPCR experiment was carried out. We collected 12 pairs of fresh tumor tissues and adjacent nontumor tissues from HCC patients. As shown in Figure 7(a), the expression level of NOL12 mRNA in HCC tissues was significantly higher than that in adjacent nontumor tissues. In addition, NOL12 expression in human liver cell L02 and four different HCC cell lines (BEL-7404, Hep3B, Huh-7, and HepG2) was examined by RT-qPCR. The results showed that compared with human liver cell, the expression level of NOL12 mRNA in HCC cell lines was significantly increased, especially in Huh-7 and HepG2 cells (Figure 7(b)). Considering that the previous data results supported the potential tumor-promoting effect of NOL12 in HCC, a series of experiments were carried out in vitro and in vivo. We selected Huh-7 and HepG2 cells with the highest expression of NOL12 in HCC cells for knockdown experiments. Two shRNAs targeting human NOL12 (shNOL12-1, shNOL12-2) were introduced into HepG2 and Huh-7 cells to detect changes in endogenous NOL12 protein. As shown in Figure 7(c), we confirmed NOL12 silencing by comparison with the corresponding negative control (shCtrl). The results of the cell proliferation assay showed that the number of cells in the NOL12-knockdown group was significantly lower than that in the shCtrl group (Figure 7(d)). The results of the MTT assay also showed that cell viability was significantly inhibited after NOL12 knockdown in vitro (Figure 7(e)), and the colony numbers of HepG2 and Huh-7 cells decreased significantly (Figures 7(f) and 7(g)). To further confirm the carcinogenicity of NOL12 in vivo, we established an HCC xenograft model by injecting Huh-7 cells with stable knockdown of NOL12 and shCtrl cells into the abdominal flanks of five-week-old male nude mice and then monitoring the growth of tumors. The results showed that tumor growth in xenograft samples formed by shNOL12-treated cells was impaired compared with shCtrl cells, and tumor volume and weight were significantly reduced (Figures 7(h) and 7(i)). In addition, the proportion of Ki67-positive cells in tumors derived from NOL12 knockdown cells was significantly lower (Figure 7(j)). Together, these findings suggested that knockdown of NOL12 significantly inhibited the growth of HCC cells in vitro and their tumor formation ability in vivo.

### 3.8. Knockdown of NOL12 Inhibits the Metastasis of HCC Cells

**Figure 8:**
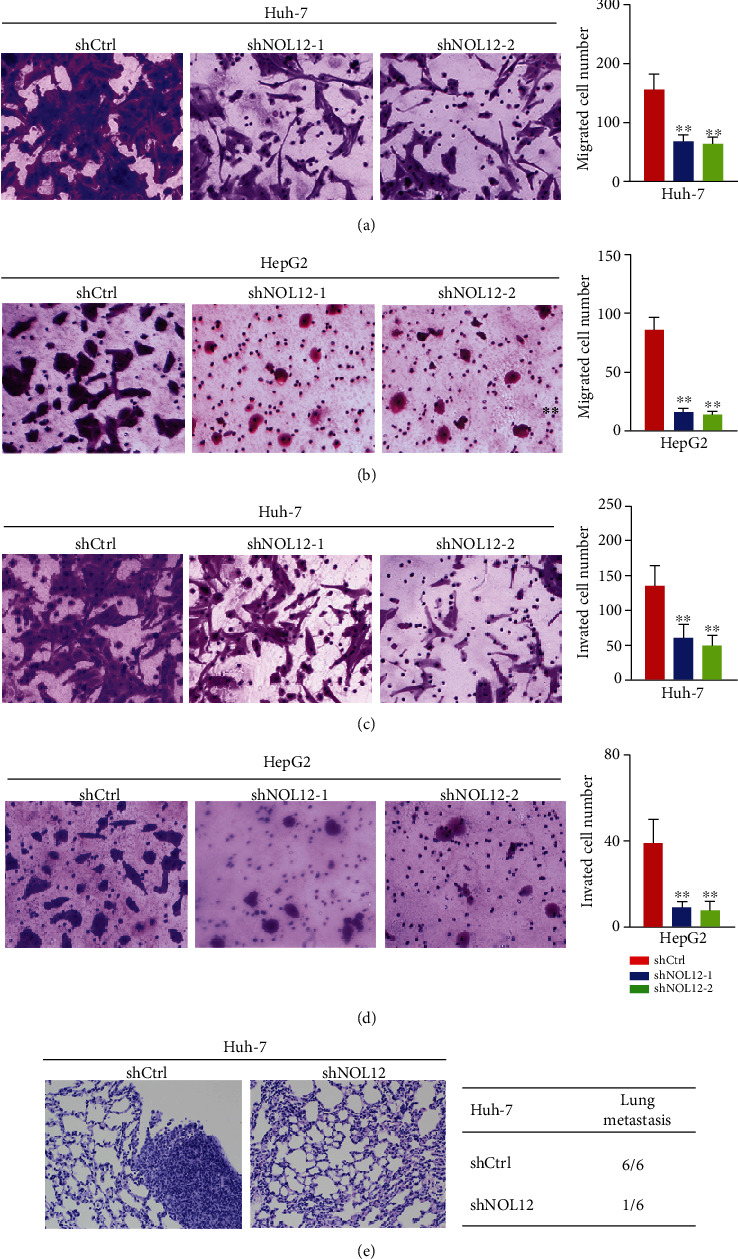
NOL12 knockdown inhibited the metastasis of HCC cells in vitro and in vivo. (a, b) Transwell migration and (c, d) Invasion assays were performed in Huh-7 and HepG2 cells. (e) Representative images of H&E staining of the lungs and incidence of lung metastasis from mice inoculated with Huh-7 cells.

Our previous results suggested that the high expression of NOL12 was related to nodal and distant metastasis in HCC patients (Figures 1(h) and 1(i)). To further determine whether NOL12 participates in the regulation of migration and invasion process in HCC cells, the transwell experiment was executed to examine the effect of NOL12 knockdown on cell migration and invasion. The results showed that knockdown of NOL12 significantly inhibited migration of Huh-7 and HepG2 cells in vitro (Figures 8(a) and 8(b)). The results of Figures 8(c) and 8(d) showed that knockdown of NOL12 also inhibited invasion of Huh-7 and HepG2 cells in vitro. Furthermore, we further study the effect of NOL12 on the metastatic ability of HCC cells in vivo by tail vein injection of Huh-7 cells. The results showed that only one tumor metastasis was observed in the lungs of the mice in the NOL12-knockdown group (1/6), while lung metastases were observed in each mouse in the shCtrl group (6/6) (Figure 8(e)). These results suggested that NOL12 played an important role in the metastasis of HCC cells.

## 4. Discussion

In recent years, although some new drugs have been approved for the treatment of HCC, the therapeutic effect of patients with advanced HCC is not satisfactory, and the 5-year survival rate has not been significantly improved [26]. How to realize the early diagnosis of HCC is still a great challenge for clinicians, so it is necessary to find new biomarkers to provide new targets for the early diagnosis and treatment of HCC [27, 28]. Previous studies have shown that RNA-binding proteins play an important role in tumorigenesis and development. Iino et al. [9] reported that the RNA-binding protein NONO can promote the proliferation of breast cancer by regulating cell proliferation-related genes and may become a potential therapeutic target for breast cancer. Li et al. [29] constructed a prognostic model that can accurately predict the prognosis of patients with lung cancer by using a variety of RNA-binding proteins. In our study, we identified a new RNA-binding protein, NOL12, that may be an oncogene of HCC.

Through bioinformatics analysis, we found for the first time that the expression of NOL12 in HCC was significantly increased, and high expression was associated with a low overall survival rate. To confirm the upregulated expression of NOL12 in HCC, RT-qPCR experiment was carried out. The results showed that the expression level of NOL12 was significantly upregulated in HCC tissues and cell lines. In addition, we also found that the high expression of NOL12 is closely related to high pathological grade, node metastasis, and advanced clinical stage. To further confirm the tumor-promoting effect of NOL12 in HCC, we carried out a series of experiments in vitro and in vivo. The results showed that NOL12 knockdown significantly inhibited growth, proliferation, migration invasion, and distant metastasis of HCC cells both in vitro and in vivo. In addition, KEGG analysis also showed that NOL12 was associated with a variety of tumor-related signaling pathways, including the MAPK, PI3K-Akt, cAMP, and Ras signaling pathways. These results strongly suggest that NOL12 can be used as a new diagnostic and prognostic marker and promote the development of HCC.

There is growing evidence that oncogenes or tumor suppressor genes can recruit different immune cells in the TME to promote or inhibit tumor progression [30, 31]. RNA-binding proteins have been shown to affect the composition of the TME through immune activation and immune regulation [10, 12]. Zhao et al. [32] reported that the RNA-binding protein SORBS2 can inhibit the TME and ultimately inhibit the metastasis of ovarian cancer by affecting the polarization of M2-like macrophages. In our study, through GO analysis, we found that NOL12 may be related to immune activation. Further research showed that NOL12 can regulate the immune activity of the TME in HCC mainly through twelve kinds of TICs. Therefore, we speculate that NOL12 can promote the immune infiltration of HCC to promote its proliferation and metastasis, but this finding needs further study to be confirmed.

Currently, access to public high-throughput gene expression datasets has contributed to the discovery of potentially reliable biomarkers of HCC [33–35]. Many research groups have found that signals based on specific gene expression can accurately predict the prognosis of patients with HCC. Dai et al. predicted the survival time of patients with HCC and the efficacy of immunotherapy by constructing an immune-related gene-based prognostic index [36]. Tang et al. constructed a prognostic signature based on four ferroptosis-related genes. This prognostic signature shows superior diagnostic and predictive performance and provides a new possibility for individualized treatment of patients with HCC [37]. Since our previous studies have shown that NOL12 can be used as a reliable biomarker for the diagnosis and prognosis of HCC, we wondered whether the signature based on NOL12-related genes can accurately predict the prognosis of patients. Here, we constructed a NOL12-related gene risk signature by LASSO regression analysis and verified it using a training set and test set. The ROC curve results showed that the risk signature has high accuracy in predicting 1-, 3-, and 5-year survival rates. Independent prognostic analysis showed that the risk signature can be used as an independent prognostic determinant of patients with HCC. In addition, the results of a stratified survival analysis showed that the risk signature could accurately predict the prognosis of patients with HCC without considering clinical factors. Because nomograms can directly show prognosis, they are widely used to predict the survival and prognosis of patients with tumors [38]. Subsequently, a nomogram was constructed based on the NOL12-related gene risk signature and other independent clinical features to predict the 1-, 3-, and 5-year overall survival rates of individual patients with HCC. The results of the calibration curve, ROC curve, and decision curve showed that the nomogram we constructed can provide clinicians with a more accurate, convenient, and practical prediction tool.

Numerous studies have shown that immune checkpoint is an important factor affecting the prognosis and treatment of advanced HCC [39]. PD-L1 expression, TIC density, TMB, and mismatch repair deficiency have been associated with the effect of ICI treatment and used to screen patients before ICI treatment [40]. In the present study, we found that the predictive power of risk signatures was related to TMB. In addition, TMB was significantly higher in the high-risk score group and predicted a poor prognosis. Therefore, we speculated that risk scores, such as TMB, may help to improve the efficacy of ICIs in patients with high risk scores. The results showed that the expression level of immune checkpoint molecules increased in patients with high risk scores, indicating that patients with high risk scores may be more sensitive to ICI treatment and benefit more. To date, this study demonstrates for the first time the important role of NOL12 in the prognosis of HCC. We believe that, for a long time, the treatment options of patients with advanced HCC are limited, and the overall survival has not been significantly improved. Fortunately, more and more drugs are used to treat advanced HCC. Among many types of drugs, ICIs may become a routine drug for patients with advanced HCC. We identified NOL12, a prognostic biomarker for HCC, and found that NOL12-related risk signature can predict the response to ICI therapy, which can provide ideas for ICI treatment for advanced HCC. However, we acknowledged that more clinical samples were needed to validate these results. In addition, more work on finding out the exact NOL12 regulatory network needs be done in the future as well as novel inhibitors targeting NOL12.

## 5. Conclusions

In conclusion, our findings revealed the expression pattern and prognostic and tumorigenic roles of NOL12 and identified NOL12 as a novel oncogene and potential therapeutic target in HCC that may be achieved by affecting the TME. In addition, the risk signature based on NOL12-related genes can guide clinicians to judge the prognosis of patients with HCC and evaluate the effect of ICI treatment.

## Data Availability

The datasets used during the current study are available from the corresponding author on reasonable request.

## Supplementary Materials

Supplementary Materials Supplementary Figure 1: distribution landscape and correlation analysis of infiltrating immune cells in tumor specimens. (A) Stacked bar chart showing the proportion of 22 kinds of immune cells in LIHC tumor samples. (B) Correlation matrix of 22 kinds of immune cell proportions. (C) Heatmap shows the differential expression of 22 kinds of immune cells in LIHC samples. Supplementary Figure 2: Kaplan–Meier survival curves for the high- and low-risk groups stratified by clinical characteristics including age (A, B), sex (C, D), grade (E, F), T stage (G, H), N stage (I, J), M stage (K, L), and clinical stage (M, N) in the complete dataset.
