# Supplementary material for: NOL12 as an Oncogenic Biomarker Promotes Hepatocellular Carcinoma Growth and Metastasis
Source: Oxid Med Cell Longev. 2022 Jun 2;2022:6891155. doi: 10.1155/2022/6891155 (PMC9184182; doi:10.1155/2022/6891155)
Supplement: Supplementary Materials — Supplementary Figure 1: distribution landscape and correlation analysis of infiltrating immune cells in tumor specimens. (A) Stacked bar chart showing the proportion of 22 kinds of immune cells in LIHC tumor samples. (B) Correlation matrix of 22 kinds of immune cell proportions. (C) Heatmap shows the differential expression of 22 kinds of immune cells in LIHC samples. Supplementary Figure 2: Kaplan–Meier survival curves for the high- and low-risk groups stratified by clinical characteristics including age (A, B), sex (C, D), grade (E, F), T stage (G, H), N stage (I, J), M stage (K, L), and clinical stage (M, N) in the complete dataset. [file 6891155.f1.zip › Supplementary Materials.pdf]

Supplementary Materials

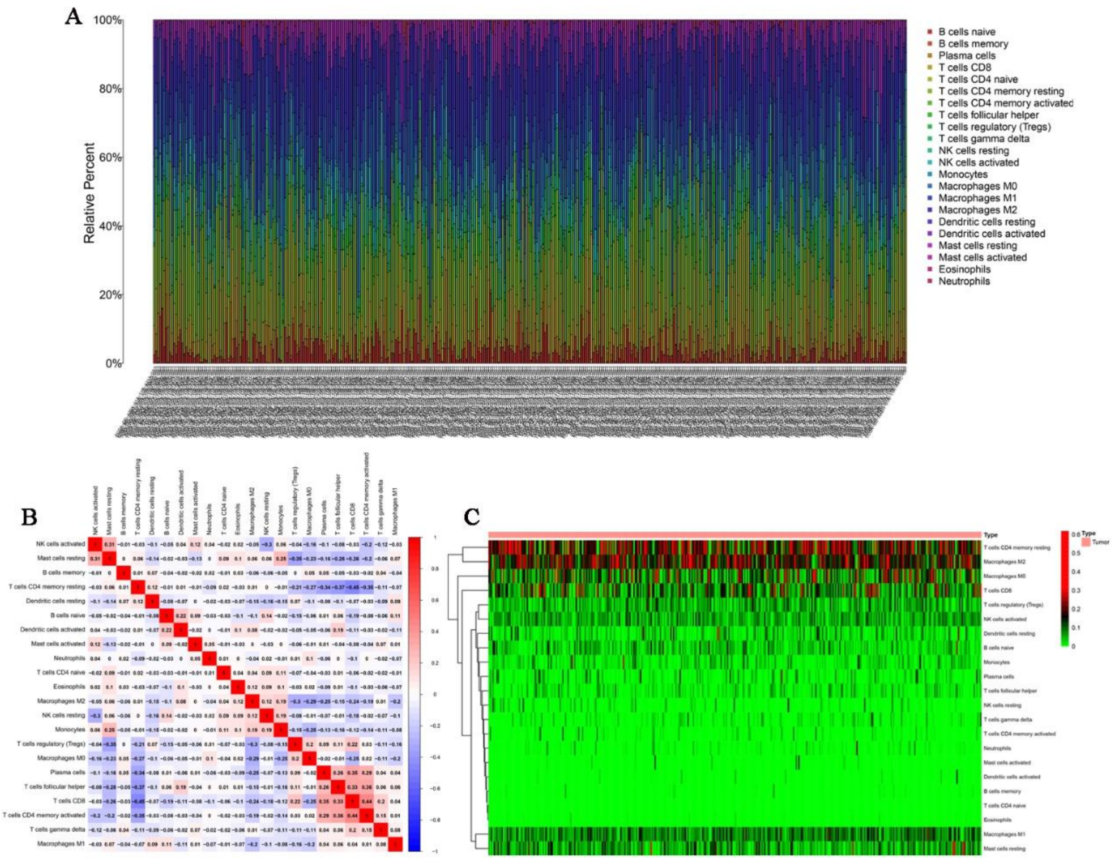

Supplementary Figure 1. Distribution landscape and correlation analysis of infiltrating immune cells in tumor specimens. (A) Stacked bar chart showing the proportion of 22 kinds of immune cells in LIHC tumor samples. (B) Correlation matrix of 22 kinds of immune cell proportions. (C) Heatmap shows the differential expression of 22 kinds of immune cells in LIHC samples.

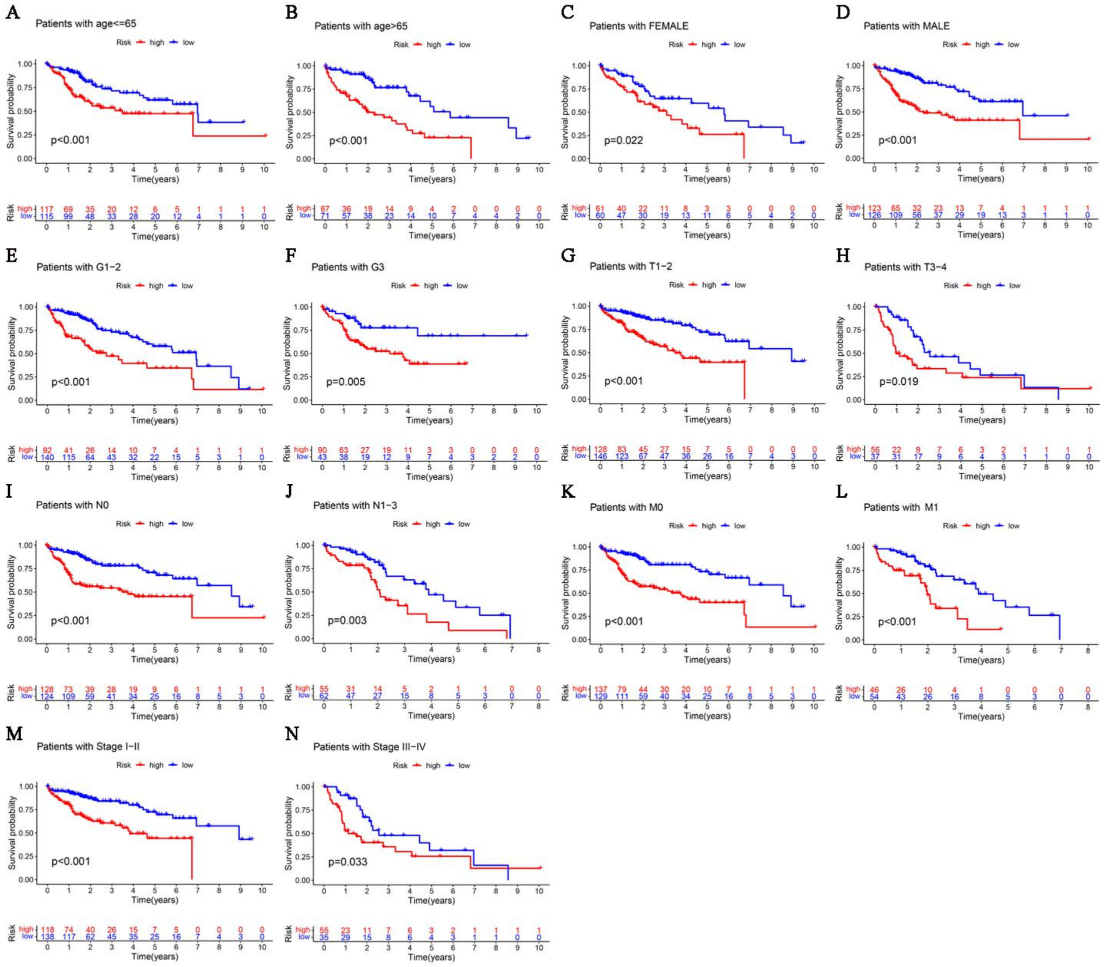

Supplementary Figure 2. Kaplan–Meier survival curves for the high- and low-risk groups stratified by clinical characteristics including age (A, B), sex (C, D), grade (E, F), T stage (G, H), N stage (I, J), M stage (K, L), and clinical stage (M, N) in the complete dataset.
